# Lipid accumulation in human breast cancer cells injured by iron depletors

**DOI:** 10.1186/s13046-018-0737-z

**Published:** 2018-04-03

**Authors:** Maida De Bortoli, Elena Taverna, Elisa Maffioli, Patrizia Casalini, Francesco Crisafi, Vikas Kumar, Claudio Caccia, Dario Polli, Gabriella Tedeschi, Italia Bongarzone

**Affiliations:** 1 Fondazione IRCCS Istituto Nazionale dei Tumori, via G. Amadeo 42, Milan, 20133 Italy; 2 Dipartimento di Medicina Veterinaria (DiMeVet), University of Milan, Milan, I-20133 Italy; 3 Molecular Targeting Unit, Fondazione IRCCS Istituto Nazionale dei Tumori, Via Amadeo 42, 20133 Milan, Italy; 4 IFN-CNR, Dipartimento di Fisica, Politecnico di Milano, Piazza Leonardo da Vinci 32, 20133 Milan, Italy; 5 Laboratory of Clinical Pathology and Medical Genetics, Fondazione IRCCS ‘Carlo Besta’ Istituto Neurologico, Via Amadeo 42, 20133 Milan, Italy; 6 Center for Nano Science and Technology at Polimi, Istituto Italiano di Tecnologia, 20133 Milan, Italy; 7 Fondazione Filarete, I-20139 Milan, Italy

**Keywords:** Breast cancer, cytoplasm vacuolation, endoplasmic reticulum stress, iron chelation, lipid droplets, macropinocytosis, mitochondria dysfunctions, methuotic cell death, hypoxia, Raman spectroscopy

## Abstract

**Background:**

Current insights into the effects of iron deficiency in tumour cells are not commensurate with the importance of iron in cell metabolism. Studies have predominantly focused on the effects of oxygen or glucose scarcity in tumour cells, while attributing insufficient emphasis to the inadequate supply of iron in hypoxic regions. Cellular responses to iron deficiency and hypoxia are interlinked and may strongly affect tumour metabolism.

**Methods:**

We examined the morphological, proteomic, and metabolic effects induced by two iron chelators—deferoxamine (DFO) and di-2-pyridylketone 4,4-dimethyl-3-thiosemicarbazone (Dp44mT)—on MDA-MB-231 and MDA-MB-157 breast cancer cells.

**Results:**

These chelators induced a cytoplasmic massive vacuolation and accumulation of lipid droplets (LDs), eventually followed by implosive, non-autophagic, and non-apoptotic death similar to methuosis. Vacuoles and LDs are generated by expansion of the endoplasmic reticulum (ER) based on extracellular fluid import, which includes unsaturated fatty acids that accumulate in LDs. Typical physiological phenomena associated with hypoxia are observed, such as inhibition of translation, mitochondrial dysfunction, and metabolic remodelling. These survival-oriented changes are associated with a greater expression of epithelial/mesenchymal transcription markers.

**Conclusions:**

Iron starvation induces a hypoxia-like program able to scavenge nutrients from the extracellular environment, and cells assume a hypertrophic phenotype. Such survival strategy is accompanied by the ER-dependent massive cytoplasmic vacuolization, mitochondrial dysfunctions, and LD accumulation and then evolves into cell death. LDs containing a greater proportion of unsaturated lipids are released as a consequence of cell death. The consequence of the disruption of iron metabolism in tumour tissue and the effects of LDs on intercellular communication, cancer–inflammation axis, and immunity remain to be explored. Considering the potential benefits, these are crucial subjects for future mechanistic and clinical studies.

**Electronic supplementary material:**

The online version of this article (10.1186/s13046-018-0737-z) contains supplementary material, which is available to authorized users.

## Background

Iron is an essential component of several cellular enzymes, such as catalases, peroxidases, cytochromes, ribonucleotide reductase, desaturases, and aconitase, which are crucial for physiological functions and have been implicated in several diseases, including cancer, because of alterations in iron metabolism [1]. Adequate iron supply is critical for various cellular processes, including DNA synthesis and cell cycle progression. Many in vitro and in vivo studies have demonstrated that compared with normal cells, cancer cells are more sensitive to iron deprivation because of their marked Fe requirements [2]. Furthermore, their strong dependency on iron is evidenced by their increased expression of transferrin receptors compared with that of normal cells. In vitro and animal studies have also indicated the antitumour activity of several iron chelators [3–5].

Clinical data support the concept that iron deficiency increases angiogenesis and causes breast cancer recurrence [6]. Specifically, iron deficiency can contribute to the high recurrence of breast cancer in premenopausal women, whereas iron load might play a role in the metastasization of breast cancer in postmenopausal women.

Iron chelators are known to induce apoptosis in several types of proliferating cells [7] and are therefore considered promising anti-proliferative agents in the treatment of human cancers. Iron chelators, such as deferoxamine (DFO), deferiprone, and deferasirox, have several advantages: they have been clinically approved for iron overload disorders [8], they have a well-studied, long-term use toxicity profile, and experimental results could be readily translated into clinical trials for cancer. Nevertheless, it is crucial to consider several side effects related to their use, including myelosuppression, hypoxia, and methemoglobinaemia, as observed from clinical trials [9].

DFO is a clinically approved non-toxic iron chelator that has been effectively used for long-term iron chelation therapy in beta-thalassemia and other iron overload disorders. DFO has also been reported to have some antitumour activity [10–13]. Novel chelators based on the di-2-pyridylketone thiosemicarbazone (DpT) scaffold, such as di-2-pyridylketone 4,4-dimethyl-3-thiosemicarbazone (Dp44mT), induce iron sequestration and also form redox-active metal complexes that demonstrate potent and selective antitumour activity [14]. Notably, Dp44mT and its analogues possess broad anti-cancer and anti-metastatic activity, in vitro and in vivo, against several aggressive solid tumours [15–20].

Relevant studies have focused on the causality between iron chelation and cancer cell death, without considering that substantial regions of cancers often grow in hypoxic conditions owing to the lack of a functional vasculature. The amount of bio-available iron is often limited in poorly vascularised areas, and the iron uptake in cancer cells is inadequate to fulfil their need. Moreover, the lack of cellular iron content has an essential role in positively regulating hypoxia-inducible factor (HIF) protein stability and therefore hypoxia mechanisms, even under non-hypoxic conditions [21]. Thus, cancer cells are more susceptible to iron depletion than non-cancer cells, a phenomenon we have termed *iron addiction*; however, it is important to note that cancer cells adapt in response to low iron levels, directly affecting cancer cell metabolism [22]. Recent findings on medulloblastoma cell lines indicate that modulation of iron-related proteins during hypoxia may increase cell proliferation as well as tumour aggression and stemness [23]. Oxygen and iron are intimately linked in producing signals through the hypoxia response pathway, and they exert considerable influence on cancer cell metabolism [24].

Based on these observations, we aimed to understand the morphological, proteomic, and metabolic effects of iron depletion in breast cancer cells with a deeper insight into the cellular effects and drawbacks of iron starvation. We combined biochemistry, microscopy, flow cytometry, and mass spectrometry-based methods to investigate the cellular and molecular events induced by DFO or Dp44mT in two human breast cancer cell lines, MDA-MB-231 and MDA-MB-157.

Cellular stress and organelle dysfunctions due to the disruption of cellular homeostasis were observed, and these events led to cell death. A dramatic increase in endoplasmic reticulum (ER) complexity, with the appearance of large vacuoles and the accumulation of lipid droplets (LDs), accompanied mitochondrial dysfunction and bioenergetics collapse and was followed by cell death and LD leakage. The results of this study are expected to provide important insights into the fundamental molecular mechanisms of the adaptation of breast cancer cells to iron limitation.

## Methods

### Cell lines and culture

MDA-MB-231 and MDA-MB-157 breast cancer cell lines were obtained from the American Type Culture Collection (ATCC, Manassas, VA, USA). They are characterised as triple-negative/basal B mammary carcinoma and considered models of triple-negative breast cancer growth and progression. Cell lines were maintained in Dulbecco’s modified Eagle’s medium (DMEM; Gibco) containing 10% heat-inactivated foetal bovine serum (FBS; HyClone), DMEM w/ 4.5 g/L glucose w/o L-glutamine at 37 °C and 5% CO_2_ in air.

### Reagents

Dp44mT and DFO were purchased from Sigma-Aldrich. Dp44mT was dissolved in dimethyl sulfoxide (DMSO) and further diluted to a final concentration of 5 μM in the culture medium, whereas DFO was diluted to a final concentration of 250 μM in the culture medium and used at a final concentration of 100 μM. Cells were incubated with either control media containing or not containing DMSO at 0.05% (*v*/v) to match the concentration of the dissolved Dp44mT or DFO.

### Assessment of antiproliferative activity

3-[4,5-dimethylthiazol-2-yl]-2,5-diphenyltetrazolium bromide thiazolyl blue (MTT) assay was performed in 48-multiwell plates containing MDA-MB-231 cells, seeded at a concentration of 1.8 × 10^4^ cells per well. After 24 h, MDA-MB-231 cells were treated with 5 μM Dp44mT or with 100 μM DFO for 120 h. Cell proliferation was assessed by MTT (3-[4,5-dimethylthiazol-2-yl]-2,5-diphenyltetrazolium bromide; thiazolyl blue) assay according to the manufacturer’s recommendations (Sigma). Absorbance intensity was quantified at 492 nm using a microplate reader (Infinite 200, Tecan). Data are shown as mean ± standard error of the mean (SEM) of quadruplicated wells and are representative of three independent experiments. Statistical tests were performed using GraphPad Prism version 5.0 (GraphPad Software Inc.).

### Sample preparation, SDS-PAGE, and immunoblotting

MDA-MB-231 and MDA-MB-157 cell lines were treated with the iron chelators for the times indicated earlier. Cell pellets were solubilised as previously described [25]. Protein concentrations were detected using a bicinchoninic acid (BCA) kit (Pierce) according to the manufacturer’s instructions, and SDS-PAGE and electroblotting were performed, as previously described [26]. The antibodies used for western blotting analyses are listed in Additional file 1.

### Mitochondrial membrane potential analysis

After treating cells with the iron chelators for the times indicated earlier, the mitochondrial membrane potential of the cells was detected using a MitoProbe™ JC-1 assay kit for flow cytometry (Thermo Fisher Scientific). Flow cytometry analyses were performed as previously described [27].

### Fluorescence microscopy

The subcellular location of various proteins was visualised by fluorescence microscopy (Eclipse E1000, Nikon Instruments, Inc.). To visualise different subcellular compartments, we used antibodies for RAB5, RAB7, LAMP1, NDRG1, and RTN4 as markers for the ER and LysoTracker red (50 nM; Life Technologies) as a marker for lysosomes. To visualise mitochondria in live cells, we stained the cells with the vital dye Rhodamine 123 (500 ng/mL; Sigma-Aldrich) for 15 min at 37 °C. To visualise nuclei in live cells, we stained the cells with the cell-permeable DNA dye 4′,6-diamidino-2-phenylindole (DAPI) (10 μM; Molecular Probes) for 15 min at 37 °C.

### Confocal microscopy

Confocal laser scanning microscopy was performed using the Leica TCS SP8 X microscope (Leica Microsystems GmbH).

### Nano-scale LC-MS/MS analysis

Cells were lysed in 50 μL of 0.1% RapidGest SF Surfactant (Waters) and diluted in 50 mM ammonium bicarbonate (pH 8.0). After reduction and alkylation, the proteins were digested with trypsin sequence grade (Roche) for 16 h at 37 °C using a protein:trypsin ratio of 20:1 [28]. LC-ESI-MS/MS analysis was performed on a Dionex UltiMate 3000 HPLC System with a PicoFrit ProteoPrep C18 column (200 mm in length and with an internal diameter of 75 μm) (New Objective). The eluate was electrosprayed into an LTQ Orbitrap Velos (Thermo Fisher Scientific) through a Proxeon nanoelectrospray ion source (Thermo Fisher Scientific) [29]. Four technical replicate analyses of each sample were performed. Data acquisition was controlled by Xcalibur 2.0 and Tune 2.4 software (Thermo Fisher Scientific).

Mass spectra were analysed using the MaxQuant software (version 1.3.0.5). Enzyme specificity was set to trypsin. Carbamidomethylcysteine was set as a fixed modification, and N-terminal acetylation, methionine oxidation, and asparagine/glutamine deamidation were set as variable modifications. The spectra were searched by the Andromeda search engine against the human Uniprot sequence database (release 29.06.2015). Protein identification required at least one unique or razor peptide per protein group. Quantification in MaxQuant was performed using the built-in XIC-based label-free quantification (LFQ) algorithm [30] using fast LFQ. The required false positive rate was set to 1% at the peptide level and 1% at the protein level. Statistical analyses [31] were performed using the Perseus software (version 1.4.0.6, www.biochem.mpg.de/mann/tools/).

Only proteins present and quantified in at least 3 out of 4 technical repeats were considered positively identified; 2478, 2488, and 2496 proteins were identified in untreated, DFO-, and Dp44mT-treated cells, respectively; 108 proteins were exclusively expressed in untreated/control cells, 54 proteins in DFO/cells, and 137 proteins in Dp44mT/cells. An ANOVA test (false discovery rate 0.05) was carried out to identify proteins differentially expressed among the three conditions: 1682 out of 2149 common proteins differed with statistical significance and were selected for further analyses. In particular, we focused on the differential proteomics between proteins expressed in untreated cells and proteins expressed in DFO- or Dp44mT-treated cells. Differential expression was considered significant if (1) a protein was present only in untreated or treated cells or (2) its normalised (according to the LFQ algorithm) intensity resulted in a statistical difference, as calculated by the Welch’s *t*-test (*t*-test cut-off at *p* value = 0.0167). The MS proteomics data have been deposited in the ProteomeXchange Consortium via the PRIDE partner repository [32] with the dataset identifier PXD007595.

### Gene ontology (GO)

The Search Tool for the Retrieval of INteracting Genes/proteins (STRING) database (version 10.5, Database issue: D412–416) [33] was used for prediction of Kyoto Encyclopedia of Genes and Genomes (KEGG) pathways [34–36]. A GO scatterplot was constructed in Excel.

### Oil red O staining

To determine the presence of LD accumulation within MDA-MB-231 and MDA-MB-157 cells, Oil Red O (Sigma-Aldrich) staining was performed. To visualise cell nuclei, samples were stained with haematoxylin (Sigma-Aldrich). Cells were imaged on a Leica DM IRB microscope (Leica Microsystems).

### Fatty acid (FA) quantification in lipid droplets

Cells were cultured in 10-cm dishes for 96 h in the presence of 100 μM DFO or Dp44mT. The presence of LDs was evaluated with Oil Red O staining. Cell debris was recovered from the plates and LDs purified by density sucrose gradient [37].

Lipids were prepared by homogenizing the samples in ethanol containing (50 ppm) butylated hydroxy toluene (BHT) to avoid oxidation [38]. A lipid chromatogram was obtained by gas chromatography–mass spectrometry (GC-MS) analysis using a Shimadzu gas chromatograph equipped with a quadrupole mass spectrometer for electron impact ionisation (GC-MS-QP2010). An SH Stabilwax DA column (30 m in length, 0.25 mm in diameter, and with a film thickness 0.25 μm) was used to separate the FA methyl ester at a flow rate of 1.0 mL/min. The injector temperature was set to 200 °C and the transfer line temperature to 280 °C. The GC oven was programmed as follows: after 2 min at 50 °C, the temperature was increased at 30 °C/min to 150 °C, then at 15 °C/min to 230 °C. The total run duration was 25 min. GC-MS analysis was conducted in the full scan mode (m/z 35–600). Qualitative analysis was based on the characteristic ions of the FA methyl esters and their relative retention times. Quantitative analysis was based on the ratio between the peak area of each FA and the corresponding internal standard peak area, using the respective standard curves.

### Raman spectroscopy

To perform coherent anti-Stokes Raman scattering (CARS) imaging, a home-built laser scanning multi-modal microscope, described previously [39], was utilised. To acquire CARS images, the treated cells were placed under the laser focus of the microscope, and the laser spot was galvo-scanned over the 50 × 50 μm^2^ sample area at two fixed pump-Stokes frequency detuning: 2850 cm^− 1^ (for lipid molecules) and 3050 cm^− 1^ (for medium and vacuoles). Spectral profiles and spatially resolved chemical maps of the pure chemical components were generated using the MATLAB tool box MCR-ALS (Multivariate Curve Resolution Alternating Least Squares) algorithm [40, 41]. We collected two images: one at 2850 cm^− 1^, indicating the symmetric vibration of CH_2_ chains in lipids, and the other at 3050 cm^− 1^, indicating the stretching vibration of hydrogen-bonded water of saline environments. The output of the software was two matrices (representing the concentration at 2850 cm^− 1^ and 3050 cm^− 1^) and two spectra (representing the two identified species). For the set-up of the figure, we have plotted these two matrices from grayscale to a single-color scale (green for lipids, blue for medium) and created a composite image thereof with the FIJI image processing package.

## Results

### Targeting iron homeostasis induces cell death by formation of vacuoles

**Fig. 1:**
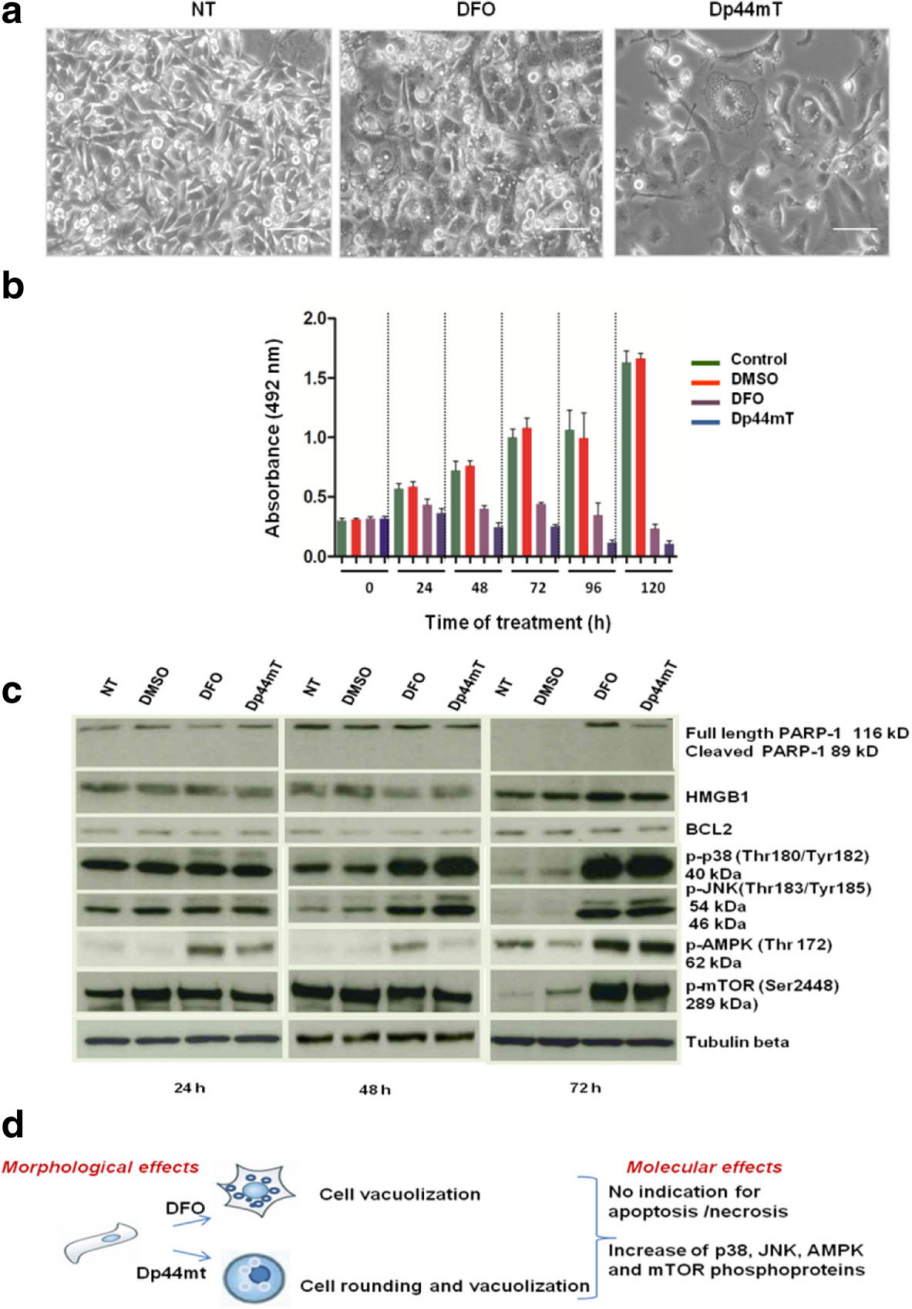
DFO/Dp44mT-induced vacuolation leads to non-apoptotic/necrotic cell death in MDA-MB-231 breast cancer cells. **a** Representative images of MDA-MB-231 cells treated with 100 μM DFO or with 5 μM Dp44mT. The cells were treated with the iron chelators for 48 h and photographed under an optical microscope. Scale bars: 100 μm. **b** MDA-MB-231 cells treated with 100 μM DFO or with 5 μM Dp44mT to determine the effects of the two iron chelators on cell proliferation. An MTT assay analysis indicated that after 24 h, cells treated with DFO or Dp44mT showed a slight decrease in cell proliferation. After Dp44mT treatment for 48 h, cell proliferation was reduced by 69%, whereas DFO treatment elicited a decrease in cell proliferation by 45%. After 72 h, Dp44mT treatment resulted in a considerable decrease in cell proliferation (75%), whereas DFO treatment reduced cell proliferation by 56%. Finally, after 120 h, proliferation was effectively blocked in MDA-MB-231 cells treated with both chelators. **c** Determination of the expressions of PARP, HMGB1, BCL-2, phosphorylated p38, JNK, AMPK, and mTOR, following treatment of MDA-MB-231 cells with DFO or Dp44mT. Cells were treated with 100 μM DFO or 5 μM Dp44mT and the protein levels were examined over a period of 24–72 h. In control samples, no PARP-1 expression was observed at 72 h. At 116 kDa corresponding to full-length PARP-1, immunoreactivity was detectable in the 24, 48, and 72 h-treated samples. No cleavage products of PARP-1 were seen at 48 h and 72 h. Weak increase in HMGB1 expression was noted in treated cells after 72 h. No change in the protein levels of BCL-2 was observed. The increased levels of phosphorylated p38, JNK, AMPK, and mTOR suggest that DFO and Dp44mT induce cell death by maintaining multiple activated pathways. Tubulin was used as a loading control. **d** Schematic representation of the effects observed with DFO or Dp44mT treatments

We observed that the treatment of MDA-MB-231 cells with 100 μM DFO or 5 μM Dp44mT induced extensive cytoplasmic modifications at different time points (24 h, 48 h, and 72 h). At 48–72 h after treatment with 100 μM DFO, the cells became flattened and showed extensive vacuolation (Fig. 1a). Cytoplasmic vacuolation began to be evident at 24 h after Dp44mT treatment; it was more pronounced after 48 h and involved extensive regions of the cytoplasm at 72 h. The MTT assay indicated that at 120 h after treatment with 100 μM DFO, MDA-MB-231 cell proliferation was completely blocked (Fig. 1b). Treatment with 5 μM Dp44mT drastically reduced cell proliferation after 48 h and killed all the cells within 120 h. Evaluation of the cell cycle distribution of MDA-MB-231 cells after treatment with DFO or Dp44mT showed that there was a slight increase in the G2 population (Additional file 2). For both treatments, the morphology of the cells during death could neither be classified as apoptotic nor as necrotic.

Western blot analyses revealed that DFO and Dp44mT induced a very low poly (ADP-ribose) polymerase-1 (PARP) cleavage and high mobility group box 1 (HMGB1) expression in cells after 72 h (Fig. 1c). B-cell lymphoma 2 (BCL-2) expression did not change significantly in response to either DFO or Dp44mT treatments. The results using biomarkers of apoptosis and necrosis indicated the irrelevant contribution of both apoptotic and necrotic cell death pathways. The levels of p-JNK and p-p38 drastically decreased after 72 h in untreated cells but persisted at high levels in treated cells; a slight increase in p-AMPK and p-mTOR levels was observed at 72 h in both untreated and treated cells. JNK and p38 signalling may be triggered by the redox state-related perturbations and may regulate expression of apoptotic downstream genes, preventing apoptosis and promoting cell survival. These results suggest the development of stress-related compensatory mechanisms to overcome cell death and permit short-term cell survival.

### Proteome analyses

**Fig. 2:**
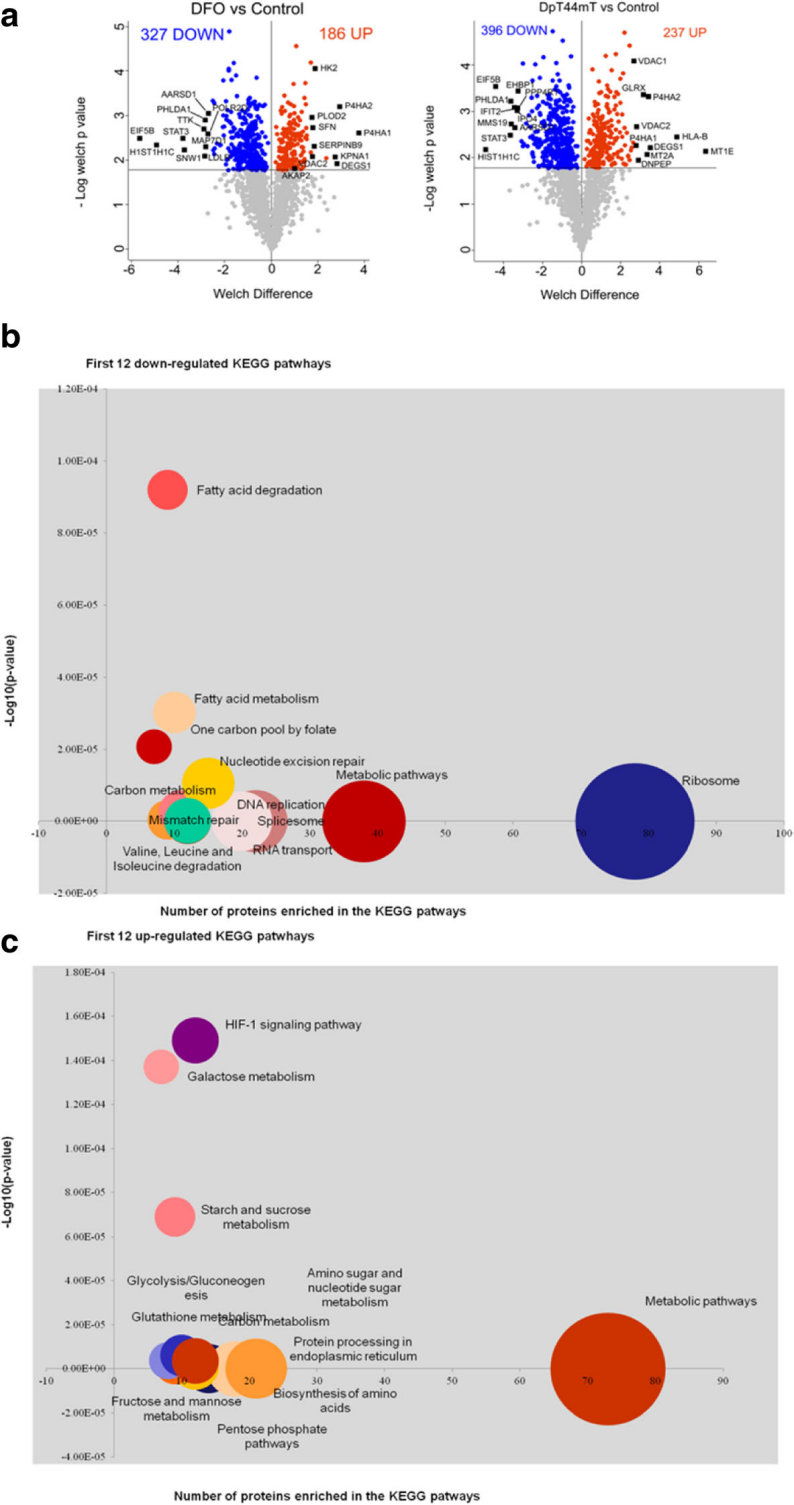
Proteomics analysis of DFO- or Dp44mT-treated cells vs untreated cells. **a** Volcano plot showing the results of differentially expressed proteins based on fold change versus *t*-test probability (Welch difference). Each protein is represented as a dot and is mapped according to its fold change on the ordinate axis (y), with *t*-test *p*-value on the abscissa axis (x). The blue and orange dots indicate up-regulated and down-regulated proteins, respectively. Grey dots have identification which did not meet the fold and *p*-value criteria. Some key molecules that changed significantly as a result of the treatments are indicated. Bubble graphs showing the top 12 down-regulated (panel **b**) and the top 12 up-regulated (panel **c**) KEGG pathway terms plotted against the -log_10_ (*p*-value) for proteins obtained from whole-cell lysate from untreated MDA-MB-231 cells compared with those from DFO- or Dp44mT-treated cells. The size of bubbles represents the number of proteins enriched for each KEGG pathway term. All pathways had a false discovery rate (FDR) < 0.001

We performed a proteomics analysis to investigate the effects of DFO and Dp44mT in MDA-MB-231 cells after treatment for 72 h, when a significant change in phenotype and morphology was observed. DMSO-treated cells were used as control. Shotgun label-free quantitative analysis was performed to examine the differences in global protein expression. After data filtering, the analysis resulted in the identification of 2478 (DMSO), 2488 (DFO), and 2496 (Dp44mT) proteins. One hundred eight proteins were exclusively expressed in untreated/control cells, 54 proteins in DFO/cells, and 137 proteins in Dp44mT/cells (Additional files 3, 4, 5, 6). An ANOVA test (false discovery rate 0.05) was carried out to identify proteins differentially expressed among the three conditions: 1682 out of 2149 common proteins differed with statistical significance and were selected for further analyses. Figure 2a shows the volcano plots summarizing the analysis of differentially expressed proteins in untreated cells in comparison with proteins expressed in DFO- or Dp44mT-treated cells.

The differentially expressed proteins for both treatments, identified in the label-free MS data, were unified and subjected to the analysis of the most significant KEGG pathways [35] using STRING software version 10 [33]. The most statistically significant decreased pathway identified by KEGG enrichment was Ribosome (#03010; *p*-value = 3.57 × 10^− 25^) with 38 proteins (Fig. 2b). However, to reflect a multi-functional stress response, several statistically significant changes in key processes and pathways were observed, referable to enhanced glycolysis, reduced translation, reduced glucose entry into the tricarboxylic acid (TCA) cycle, up-regulated glutathione metabolism and glutaminolysis, and changes in mRNA, amide, and lipid metabolism. Significant increases of the amino acid metabolism also took place as well as reduction–oxidation (redox) homeostasis and NAD metabolism. Taken together, these changes indicated a metabolic adaptation similar to that sustaining cancer cell survival during hypoxia [42].

Interrogation of the Ingenuity Pathway Analysis software highlighted that the canonical and toxicity pathways were altered by DFO or Dp44mT (Additional file 7). The analysis of pathways, conducted by combining all the up- and down-regulated proteins, indicated that EIF2 signalling, eIF4-p70S6K signalling, mTOR signalling, oxidative stress, glycolysis, gluconeogenesis, and protein degradation mediated by ubiquitination were the most common and important pathways affected by DFO and Dp44mT. Tox list analysis indicated that renal necrosis/cell death pathway, mitochondrial dysfunction, HIF signalling, oxidative stress, protein degradation mediated by ubiquitination, and fatty acid (FA) metabolism were the most affected pathways.

The observed changes can be largely explained by the induction of severe defects in the Fe–S cluster biogenesis/repair, which can profoundly decrease the activities of respiratory complexes, ribosome biogenesis, and the TCA cycle, affecting energy production and increasing oxidative stress. The defects in the Fe–S cluster biogenesis might also affect cytosolic aconitase activity and therefore citrate metabolism, which can disrupt the balance between glycolysis, FA biosynthesis, and purine catabolism pathways. An increase in glutamic-oxaloacetic transaminase (GOT) 1 activity was observed, indicating a possible increase in glutamine (Gln) metabolism with an apparently high uptake of Gln through up-regulation of sodium-dependent neutral amino acid transporter type 2 (SLC1A5). Along with the metabolic modifications in the treated cells, we also regarded the reduction of FA synthase (FASN) as possibly indicative of the reduction of lipid synthesis; the up-regulation of perilipin (PLIN) 2 and PLIN3 was predictive of LD accumulation [43] and the variation in desaturase levels (decreases in FA desaturase (FADS) 2 [44] and increases in D4-dihydroceramide (DhCer) desaturase (DEGS) 1 [45]).

The KEGG list of up-regulated pathways included the amino sugar and nucleotide sugar metabolic pathways (Fig. 2b and c). The hexosamine biosynthetic pathway (HBP) is frequently up-regulated in cancer cells with a mesenchymal phenotype [46]. It is required to sustain sufficient growth factor signalling and Gln uptake to support cell growth and survival. Glucose and Gln are both essential for the first committed step and rate-limiting step of the HBP: the conversion of fructose-6-phosphate (Fru-6P) to glucosamine-6-phosphate is required for the glycosylation of the proteins controlling the O-GlcNAcylation of nuclear and cytoplasmic proteins. Hypoxic cancer cells have been shown to contain elevated levels of the HBP genes, including Gln–fructose-6-phosphate transaminase 1 (GFPT1) (which was overexpressed in our study) and GFPT2, and overall O-GlcNAcylation [47]. Taken together, these data support a model in which by inducing a hypoxia-like metabolic reprogramming, iron chelation could solicit an HBP-based survival program that can help establish a short-term survival strategy in MDA-MB-231 cells to avoid apoptosis.

### Blocking of autophagy

**Fig. 3:**
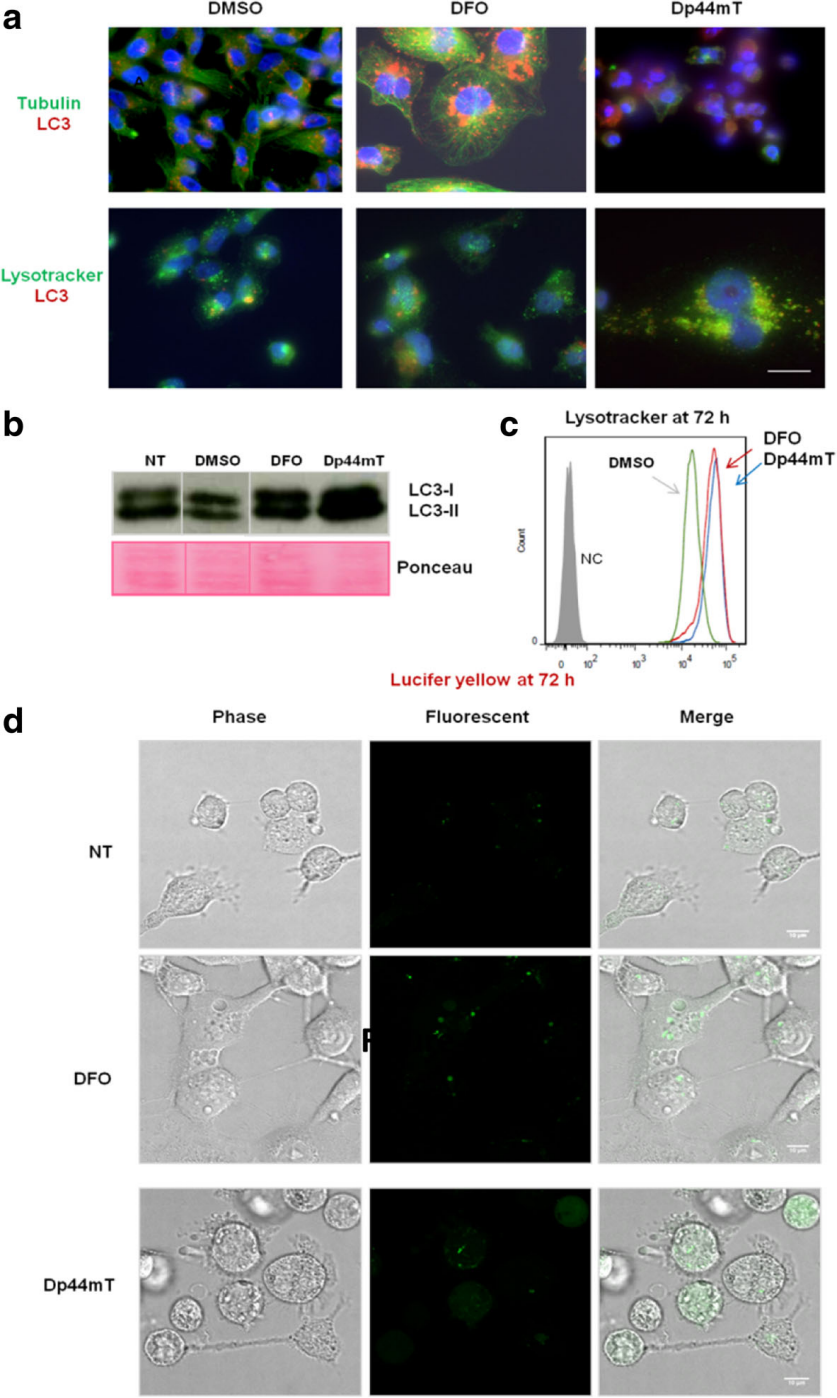
Vacuoles remain distinct from autophagosomes. **a** Immunofluorescence localisation of LC3 in vacuolated MDA-MB-231 cells. Cells were treated with DFO or Dp44mT for 48 h, immunostained with anti-LC3 (red) and with anti-tubulin (green) antibodies, and then analysed by confocal microscopy. Blue, DAPI. The cell population shifts in size and granularity and becomes heterogeneous after DFO or Dp44mT treatments. This is an excerpt of the results obtained from several (at least three) experiments. There was little overlap between the phase-lucent vacuoles and compartments labelled with markers for lysosomes (LysoTracker) or autophagosomes (LC3II), suggesting a defect at the late endosome-lysosome boundary. **b** Western blotting analysis of LC3-I and LC3-II expression in treated cells for 48 h and the relative Ponceau-stained filter used as a loading control. **c** Lysotracker staining analysis by flow cytometry analysis. Results are expressed as mean of the far red (APC-A) fluorescence intensity or forward-scatter (FSC-A) factors (x-axis) and of the side-scatter (SSC-A) factor (y-axis), i.e. granularity and size, respectively, of untreated and DFO- or Dp44mT-treated MDA-MB-231 cells for 48 h, showing the increased SSC-A in DFO-treated cells. DMSO-treated cells have lower signal intensity. **d** Confocal microscopy analysis of induced vacuoles sequestering lucifer yellow (LY). Differential interference contrast (DIC) images and LY fluorescence (green) of control, DFO-, and Dp44mT-treated cells. Not all vacuoles were sufficiently acidic to massively incorporate LY. Dp44mT-treated cells show a diffuse staining pattern throughout the cytoplasm, which could correspond to some degree of intracellular membrane permeabilisation. This is an excerpt of the results obtained from several (at least three) experiments. Scale bars: 100 μm

In principle, iron depletion can induce the activation of pathways linked to the activation of autophagy. The vacuoles formed in response to iron depletion in this study were similar to the lysosomal structures reported in several autophagic circumstances [48–52]. Thus, we investigated the possibility that the vacuoles observed in our study were also associated with autophagy. Microtubule-associated protein light chain 3 (LC3) and other members of its family are retained inside autophagosomes. Thus, LC3 is commonly used as a marker for identifying autophagosomes [53–56]. Positivity for LC3 punctae increased with both DFO and Dp44mT treatments; however, LC3 was also present in untreated cells (Fig. 3a, b). These data were further confirmed by western blotting analysis, with only a slight increase in LC3-II lipidated isoforms in treated cells (Fig. 3c).

We investigated lysosomal conditions in MDA-MB-231 cells treated with DFO or Dp44mT using lysosomotropic metachromatic fluorochrome (LysoTracker) that emits a red fluorescence at high concentrations within intact lysosomes [57]. Quantitative data from flow cytometry indicated that fluorescence increased after DFO and Dp44mT treatments (Fig. 3d). The cells exposed to Dp44mT accumulated LysoTracker in the cytoplasm, indicating a certain amount of lysosomal membrane permeabilisation. Control cells and DFO-treated cells displayed a discrete punctate fluorescent staining pattern in perinuclear regions with no fluorescence in the cytoplasm, whereas Dp44mT-treated cells displayed vacuole staining and some membrane leakage, as indicated by the diffusion of fluorescence into the cytoplasm. However, the LysoTracker staining merged with LC3 staining only partially.

Lucifer Yellow (LY) is a marker of fluid-phase pinocytosis that accumulates in secondary lysosomes and cellular membranes are impermeable to LY [58]. We loaded MDA-MB-231 cells treated for 48 h with DFO or Dp44mT with LY in the final 16 h (Fig. 3e). Control cells and DFO-treated cells had clear punctuate labelling, possibly corresponding to the lysosomal vesicles; however, the large vacuoles did not fluoresce or fluoresced scarcely. Dp44mT-treated cells also had scarce LY-positive punctate vesicles, but often, the fluorescence was more diffuse throughout the cell body, indicating leakage of LY from the secondary lysosome into the cytosol.

These findings strongly suggest that the vacuoles do not merge with lysosomes or autophagosomes and do not have acidic content. It was clear that most vacuoles were initially derived from another source, and one possibility was that they originated from non-functional late endosomes.

### Iron depletion-induced vacuolisation of cells results from ER dilation

**Fig. 4:**
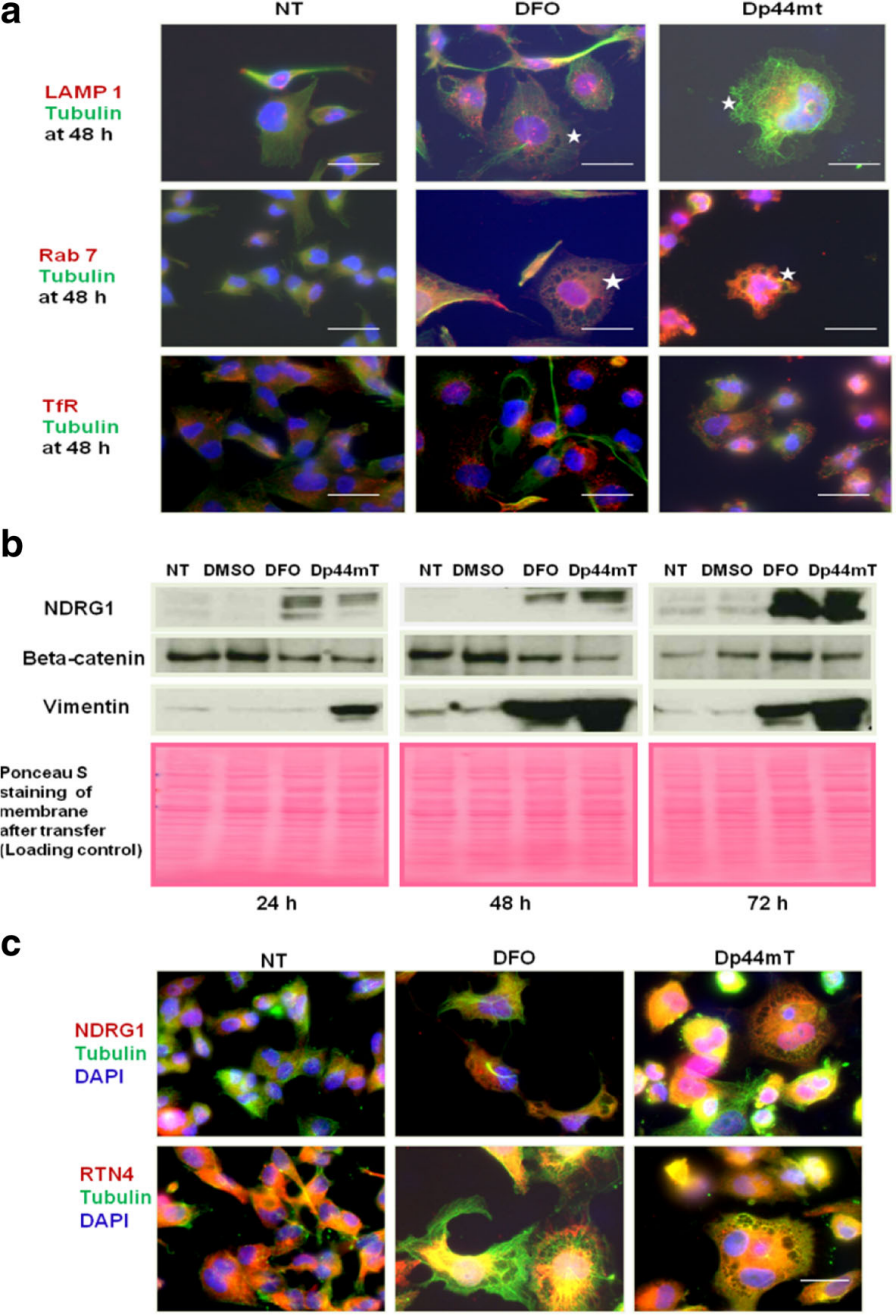
Cell phenotype represents marked dilatation of the rough endoplasmic reticulum (ER). **a** Cells were treated with DFO or Dp44mT for 48 h, processed for immunofluorescence, and then observed under a fluorescence microscope. The vacuoles were identified to be positive for late endosome/macropinosome markers RAB7 and LAMP1. Stars indicate the localisation of RAB7 and LAMP1 surface proteins in the membranes of vacuoles. TfR was used as a marker for endocytosis. **b** Western blotting analysis of the expression of the epithelial to mesenchymal transition markers NDRG1, beta-catenin, and vimentin. The protein lysates were obtained from cells treated with DFO or Dp44mT for the times indicated. **c** Localisation of NDRG1 and RTN4 in vacuolated MDA-MB-231 cells. Cells were treated with DFO or Dp44mT for 48 h, processed for immunofluorescence, and then observed under a confocal microscope. Cells were immunostained with NDRG1 and RTN4 (red) and anti-tubulin (green) antibodies and then analysed by fluorescence microscopy. Blue, DAPI. Scale bars: 100 μm

Immunofluorescence analysis for the lysosomal-associated membrane protein (LAMP) 1 showed an increase in total LAMP1 labelling in treated cells (Fig. 4a). Vacuoles were partially covered by LAMP1. In immunofluorescence microscopy experiments, DFO- and Dp44mT-induced vacuoles in MDA-MB-231 cells were well distinguished using anti-tubulin and anti-RAB 7 antibodies (Fig. 4b). Proteomics analysis showed that both tubulin and RAB 7 were up-regulated (Additional files 4, 6). Additionally, the endosomal marker, transferrin receptor (TfR), was also up-regulated by DFO and Dp44mT treatments, as determined by proteomics analysis (Additional files 4, 6) and immunofluorescence microscopy experiments (Fig. 4).

These results indicated that vacuoles represented enlarged late endosomal structures. Large intracellular vacuoles are membranous structures derived from intracellular organelles that are frequently formed in response to cell stress [59]. These observations suggested a possible, previously unidentified link between ER expansion and the adverse effects of DFO and Dp44mT.

N-myc downstream-regulated gene 1 (NDRG1) is a stress-responsive protein [60]. Immunofluorescence microscopy and western blotting data showed an increased expression of the metastasis suppressor, NDRG1, after treatment with DFO or Dp44mT. NDRG1 was diffusely expressed in the cytoplasm and nucleus in untreated cells; the treatments caused the redistribution of NDRG1 with a punctate deposition around the vacuoles. NDRG1 partly co-localised with cytoplasmic and perinuclear tubulin (Fig. 4c), which is in accordance with previous results [61], but contradicts an earlier interpretation that epithelial–mesenchymal transition (EMT) is paralleled by a down-regulation of NDRG1 expression [62]. Along with NDRG1 overexpression, we observed the loss of cell–cell junctions and gain of mesenchymal characteristics via overexpression of the mesenchymal marker, vimentin [63], and decreased expression of the epithelial marker, beta-catenin (Fig. 4b).

To gain insights into the diversity of vacuolar structures formed from the expansion of the ER—for instance, if they are flat and cisternal or highly curved and tubular—we examined the distribution of reticulon 4 (RTN4), an ER-shaping protein, that was observed to be up-regulated in our proteomics analysis (Additional files 4, 6). Figure 4c illustrates the sharp demarcation of the tubule-to-vacuole change at the cell periphery, as displayed by immunostaining of RTN4. A relatively planar network could be stained in the thin periphery of the cells, but a dense ER-associated intensity was seen in the perinuclear regions with the increase in vertical space. This pattern has been described as providing an increased surface for modulating lipid synthesis or protein folding [64]. Such a tubular membrane reservoir may also be needed to facilitate the availability of ER membranes for modulating interactions with other organelles and intracellular entities, such as mitochondria, LDs, or endocytic compartments [64].

Following the proteomics analysis, we noted a strong increase in PLINs 2 and 3 (Additional files 4, 6), which are considered LD scaffold proteins that serve as critical determinants of LD formation and cellular lipid metabolism within mammalian cells [65, 66]. In contrast, septins regulate the fluid-phase cargo traffic to lysosomes by promoting macropinosome maturation, and the fusion with endosomes/lysosomes were down-regulated (Additional files 3, 5).

### Disruption of mitochondrial membrane potential (Δψm)

**Fig. 5:**
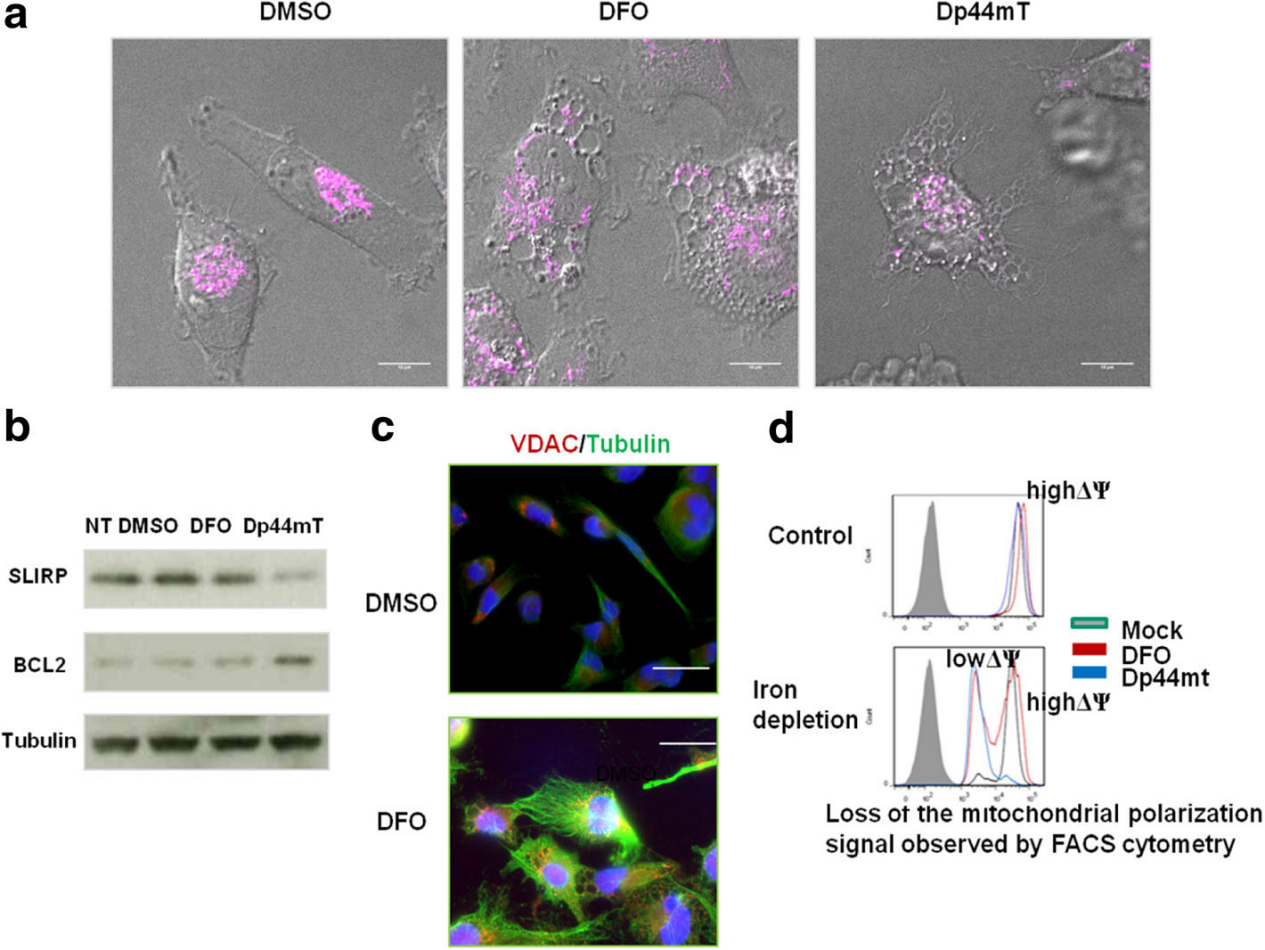
Mitochondrial alterations in MDA-MB-231 cells induced by DFO or Dp44mT treatment. **a** Using confocal microscopy, alterations in mitochondrial morphology were evaluated using Rhodamine 123 (pink). Differential interference contrast (DIC) images and Rhodamine 123 fluorescence of control (left), DFO- (centre), and Dp44mT-treated cells (right). Only representative merged images are shown. The treatment with 100 μM DFO or 5 μM Dp44mT causes ER expansion with vacuolation, which is accompanied by breakage of the mitochondrial network. **b** Western blot analysis of SLIRP and BCL-2 expression in total lysates from untreated (NT or DMSO) and treated (DFO or Dp44mT) MDA-MB-231 cells. Tubulin was used as a loading control. **c** Cells were observed under an Olympus Fluo View 1000 fluorescence microscope. MDA-MB-231 cells, untreated or treated with DFO, were labelled with an anti-VDAC antibody (red) followed by appropriate secondary antibodies, anti-tubulin (green), and DAPI (blue). The cells were observed under an Olympus Fluo View 1000 confocal microscope. Only representative merged images are shown. Scale bars: 100 μm. **d** Fluorescence-activated cell sorting (FACS) cytometry assessing mitochondrial depolarisation induced by DFO and Dp44mT

Mitochondria perform important catabolic reactions, such as the Krebs cycle and oxidative phosphorylation, which enable efficient ATP generation required for many cell functions. Mitochondria physically interact with the ER, and this interaction is important for both lipid synthesis and calcium handling [67]. During nutrient starvation, mitochondria elongate in order to preserve their metabolic function. To determine possible changes in mitochondrial distribution associated with ER expansion, we performed immunostaining experiments with Rhodamine 123. The Rhodamine 123 staining pattern clearly indicated a change in the mitochondrial network morphology after treatment with both chelators (Fig. 5a), possibly predicting the blockage of mitochondrial fission. In untreated cells, mitochondria were distributed throughout the cytosol, with a tendency to aggregate around the nucleus, forming a network. Following DFO or Dp44mT treatment, we observed fragmented mitochondria around ER vacuolar structures. Anti-VDAC1 staining confirmed this mitochondrial pathway (Fig. 5c). DFO and Dp44mT caused significant network fragmentation that typically occurs in response to severe stress, impaired oxidative phosphorylation, and cellular dysfunction [68].

The change in Rhodamine 123 fluorescence intensity reflects the change in relative levels of ΔΨm or reactive oxygen species (ROS); consistent changes were observed in both treatments but with varying degrees. Both treatments produced a bi-parametric population represented by the intensity of the signals corresponding to the *x*-axis evaluated as a function of time (Fig. 5d). Mean fluorescent intensity of the treated cells decreased with respect to that of untreated cells.

The expression of the mitochondrial marker SRA stem-loop interacting RNA-binding protein (SLIRP) decreased at 72 h (Fig. 5b). SLIRP is required for the correct binding of mitochondrial mRNAs with the mitochondrial ribosome to enable efficient translation [69]; its decreased expression possibly indicates a down-regulation of mitochondrial gene expression.

### Iron chelation induces accumulation of fluid-filled vacuoles and LDs

**Fig. 6:**
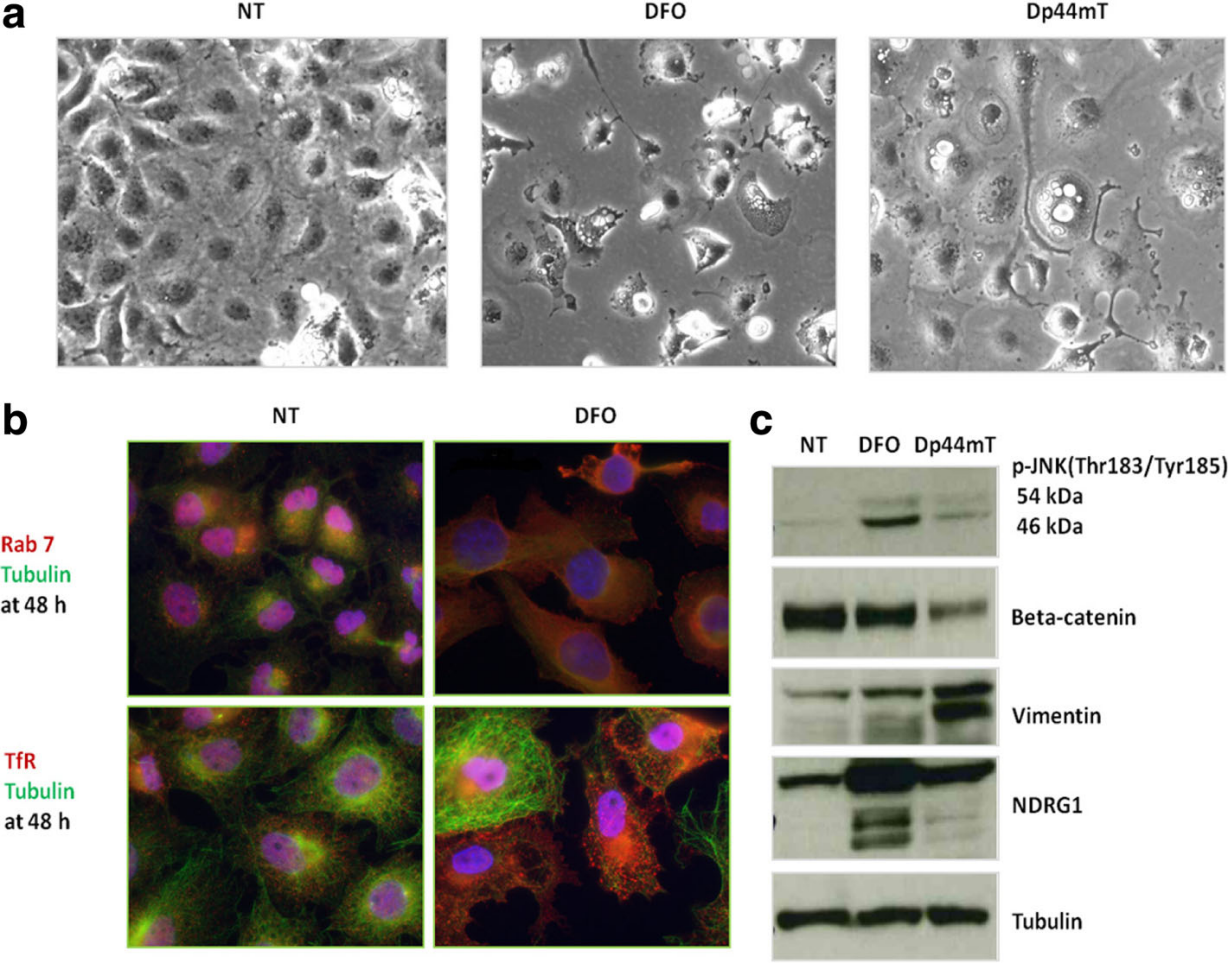
Modifications of morphology and protein expression observed in the cell line MDA-MB-157 during iron depletion. **a** MDA-MB-157 cells were treated with 100 μM of the iron chelator DFO for 72 h and photographed under an optical microscope. Scale bars: 100 μm. **b** MDA-MB-157 cells, untreated or treated with 100 μM DFO for 48 h, were labelled with anti-Rab 7 or anti-TfR antibody (red) followed by appropriate secondary antibodies, anti-tubulin (green), and DAPI (blue) and observed under an Olympus Fluo View 1000 fluorescence microscope. Only representative merged images are shown. Scale bars: 100 μm. **c** Western blot analyses of DFO- or Dp44mT-treated cells showing increased levels of phosphorylated JNK, beta-catenin, vimentin, and NDGR1. Tubulin was used as a loading control

**Fig. 7:**
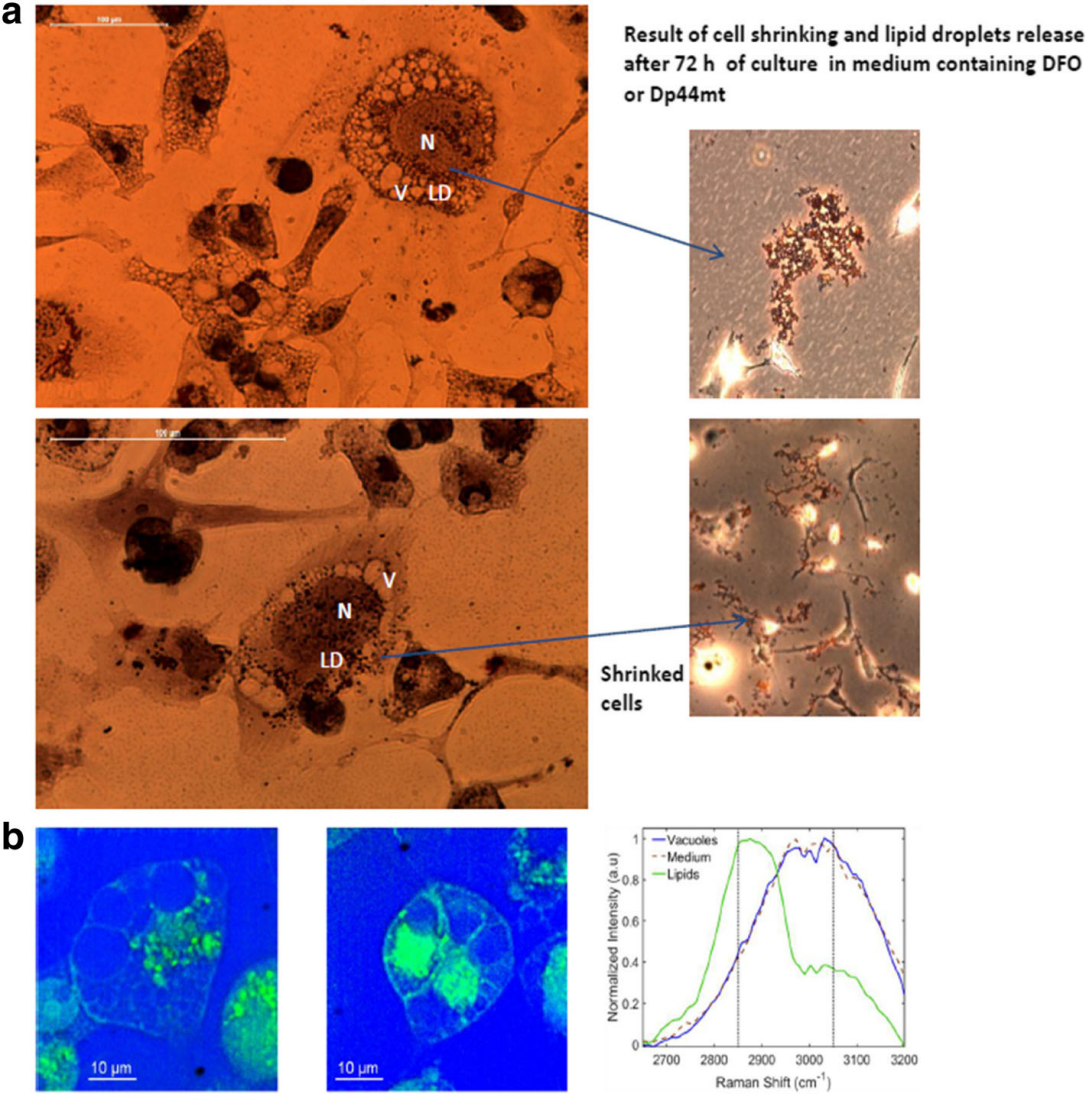
Oil Red O staining and label-free CARS imaging of MDA-MB-231 cells after DFO treatment. **a** Oil Red O staining of MDA-MB-231 cells treated with DFO for 48 h. Images on the left show cells under a microscope at magnifications of 200× and 400×. Cells were stained with Oil Red O and counterstained with haematoxylin to show nuclei (N). The cells adopted a round morphology and accumulated large cytoplasmic vacuoles (V) in which accumulation of lipids (LD) was interspersed. Images on the right show examples of how cell morphology evolves after 72 h of treatment. Cells shrink before dying with a consistent release of aggregates of floating lipid droplets. **b** CARS composite images of MDA-MB-231 cells treated with 100 μM DFO for 72 h. Panels show the images of lipids in green (2850 cm^− 1^) and of the vacuoles and medium (3050 cm^− 1^); CARS spectra are also reported

The same modifications in phenotype, cell behaviour, and some representative molecular patterns observed in MDA-MB-231 were also found in the DFO- or Dp44mT-treated MDA-MB-157 breast cancer cell line (Fig. 6). This consistency in the results indicated the existence of a possible general correlation between iron depletion and the described effects. To further understand the observed modifications, we performed further determinations. As described earlier, after treatment with DFO or Dp44mT, large, swollen vacuoles appeared suddenly in MDA-MD-231 cells during the period from 48 to 72 h, and the treated cells showed a massive accumulation of LDs that stained positively for Oil Red O in living as well as dying cells (Fig. 7). The LDs could be seen interspersed between the clear vacuoles. The treated cells exhibited considerably increased numbers of LDs, which is in agreement with the increased expression of PLIN2/PLIN3 (Additional files 4, 6).

Non-linear light microscopy techniques, such as coherent anti-stokes Raman scattering (CARS) microscopy, was used to determine the vacuolar content and to visualise LDs within a single living cell [39]. The results showed that cells incorporate large amounts of extracellular material that accumulate into large and transparent vacuoles. Therefore, the expansion of the ER lumen to form vacuoles is possibly due to the scavenging of extracellular macromolecules that are needed to sustain cell survival in a nutrient-depleted environment. The swollen ER network accumulates lipids to form ER-derived LDs generated by budding mechanisms.

Finally, when cell swelling occurred 72 h after treatment with the chelators, vacuoles largely disappeared, and droplets were extruded by the cells. Many of these droplets converged to form branched aggregated of LDs (Fig. 7).

**Fig. 8:**
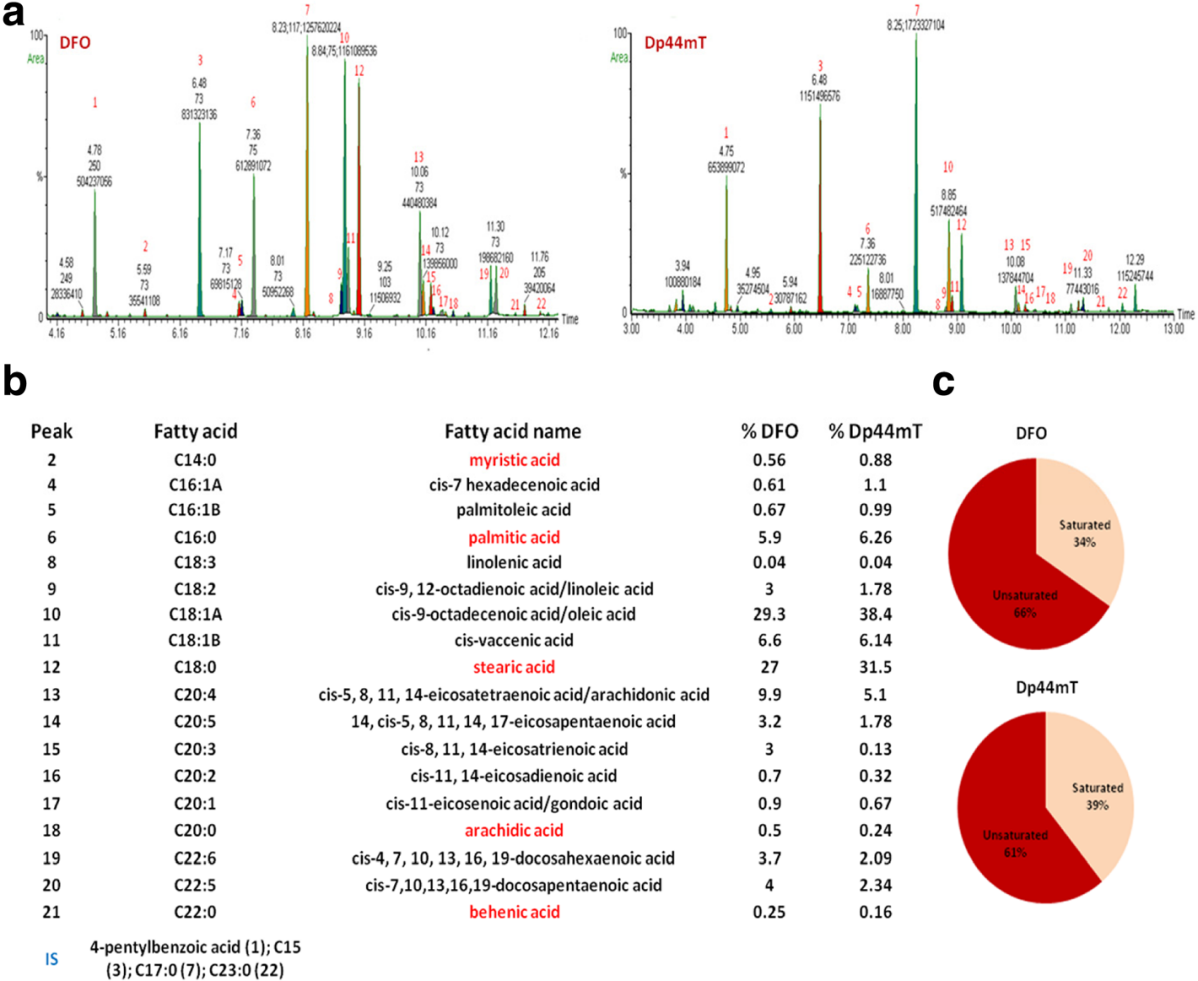
FA quantification by GC-MS. **a** GC-MS analysis of FAs obtained from LDs. FAs were extracted from LDs purified from DFO- and Dp44mT-treated cells and subjected to GC-MS analysis as described under ‘Materials and Methods’. The major FA subtypes in the LDs were oleic, stearic, and arachidonic acids. **b** Peak number, name of the FA, and FA quantification reported as peak area percentage. **c** Proportions of unsaturated and saturated FAs

LDs are the intracellular sites for neutral lipid storage with total FA composition that can vary notably. The GC-MS analysis of LDs derived from both DFO- and Dp44mT-treated cells showed that the major FAs present were oleic acid (29.3 and 38.4%, respectively), stearic acid (27 and 31.5%, respectively), arachidonic acid (ARA, 9.9 and 5.1%, respectively), and palmitic acid (5.9 and 6.26%, respectively) (Fig. 8). Notably, ARA can be metabolised into important lipid mediators and signalling molecules. Myristic acid is a saturated FA produced by FASN and was observed to be down-regulated by the treatments, representing only 0.56 and 0.88%, respectively. Oleic acid, the most abundant FA among the analysed LDs, is a monounsaturated FA synthesised by the introduction of a *cis* double bond at the Δ-9 position of stearic acid (C18) [70]. This oxidative conversion of stearic acid to oleic acid is catalysed by the NADH-dependent stearoyl-CoA desaturase (SCD), which was not observed to be differentially expressed in our proteomics analysis (Additional files 3, 4, 5, 6), and that possibly explains the reduced SCD activity in hypoxia. The lipogenic acetyl-CoA carboxylase (ACACA) was also down-regulated (Additional files 3, 5).

Previous studies have demonstrated that reduced SCD activity in hypoxic cells was associated with the scavenging of exogenous monounsaturated FAs to maintain a viable desaturation index [71]. Otherwise, a sustained ER stress was observed. In contrast, we observed that the majority of FAs in LDs were unsaturated; the desaturation index was very high (34% saturated vs 66% desaturated for DFO-treated cells and 39% saturated vs 61% desaturated for Dp44mT-treated cells), and oleic acid (29.3 and 38.4%, respectively) was more abundant than stearic acid (27 and 31.5%, respectively).

The role of LDs was not investigated in the present study; our results reveal a link between iron depletion, reduced mitochondrial activity, and LD accumulation and show that the mono-saturated oleic acid is the most abundant FA present in the LDs.

## Discussion

Iron is an essential nutrient required for many biosynthetic and metabolic processes, such as haem and sulphur–iron cluster biosynthesis, oxygen transport, mitochondrial respiration, lipid desaturation, and DNA replication [72]. Compared with normal cells, cancer cells require a large amount of iron for several essential processes. Iron bio-availability can be affected by the lack of functional vasculature in tumours, resulting in a hypoxic/anaemic tumour microenvironment. Iron affects the HIF pathway, and iron deficiency can produce responses similar to hypoxia, as observed from the effects of iron chelation in cell culture and in humans [73]. However, the effects of iron deficiency on hypoxia behaviour and signalling mechanisms have not been completely understood.

Our study provides novel insights on how iron deprivation modulates the cell structure and the proteomic and metabolic pathways in breast cancer cells. Prolonged iron deficiency in cultured tumour cells induces significant changes in cellular phenotypes and mesenchymal markers and an adaptive cellular response similar to that induced by hypoxic stress, prior to killing the tumour cells [74]. HIF-1 was up-regulated in MDA-MB-231 cells (Additional files 4, 6) after treatment with iron chelators, sustaining EMT and hypoxia programs; mesenchymal marker genes, NDRG1 and vimentin, increased in parallel with the decrease in E-cadherin expression and the increase in CD44 and CD166 expression, which are well-known cancer stem cell markers [75] (Additional files 4, 6). Remodelling of iron-starved cells showed a marked reduction in translation and the increase of redox pathways, along with perinuclear mitochondrial clustering disruption, depolarisation, and a hypoxia-like reprogramming of metabolism. The reprogramming suggests an adaptation of the cancer cells to iron deprivation, which is accompanied by the augmentation of mesenchymal characteristics.

The expanded ER formed large vacuoles and a complex network of membranes around the nucleus. Coalescent spherical units of translucent material around the nucleus were stained with lipid dyes such as Oil Red O and observed using Raman spectroscopy. These structural modifications occurred in parallel with the increased expression of ER markers (NDRG1 and RTN4) and LD-associated proteins (PLIN2/PLIN3). Using Raman spectroscopy, we could also determine that the vacuoles contained sacs of fluid-filled extracellular medium. These modifications increased with time of treatment with DFO or Dp44mT and were tolerated by the cells before they underwent cytoplasmic collapse, succumbing to a non-apoptotic and non-autophagic type of death.

Thus, under iron deprivation, breast cancer cells exhibited a large amount of fluid-filled vacuoles and lipid accumulation. A type of extensive vacuolation followed by cell death, known as methuosis, or death by macropinocytosis has been previously described as an efficient method for inducing cell death in different types of cancers [76, 77]. Proteomics analyses of pre-death cells clearly indicated a striking metabolic plasticity, based on the scavenging of nutrients destined for short-term cell survival.

By recycling nutrients from intracellular macromolecules, autophagy represents an important cellular strategy to sustain viability during periods of limited nutrient availability [78]. We observed a blocking of autophagy and possible loss of cell viability, which may be derived from the cytoplasmic hyper-vacuolisation associated with the loss of metabolic capacity (as indicated by the decrease in mitochondrial membrane potential) and plasma membrane integrity, until osmotic pressure permitted. Cells formed non-acidic and non-autophagic vacuoles, with massive cytoplasmic vacuolations developing from the dilation of the ER lumen. The formation of intracellular vacuoles and subsequent cell death indicated that the events occurring in the ER initiate this destructive pathway. This massive dilation of the ER finally culminates in a paraptosis-like cell death [3], releasing LDs into the extracellular environment. Consequently, in vivo anaemic/hypoxic regions in tumours with poor iron availability may be considered to be at risk for dissemination of possible bioactive vesicles.

The association between LD metabolism and cancer cell survival and metabolism is unknown. LD accumulation probably results from the increased lipid scavenging activity in MDA-MB-231 cells, rather than augmented lipogenesis [79]. In accordance with this view, we observed a reduction in FASN levels, which could limit the initial step in FA biosynthesis, and the KEGG indication of an increase in lipid degradation, suggesting that cancer cells scavenge lipids from the extracellular environment. Importantly, hypoxic cells exhibit increased uptake of unsaturated lipids from their environment, thus bypassing the requirement for FA desaturation [80]. In fact, FA desaturases, mainly SCD, are among the most sensitive targets of oxygen and iron starvation [81]. We observed only a decrease in delta-6 desaturase (FADS2) abundance in treated cells accompanied by the increase in LD accumulation. Based on this result, we expected a change in lipid composition with an increase in saturated FAs. Instead, a large amount of monounsaturated oleic acid was observed in DFO-and Dp44mT-induced LDs (29.3 and 38.4% of total FAs), which is generally regarded as cytoprotective [82]. Although an abundance of saturated stearic acid (27 and 31.5%), possibly due to the reduction of desaturase functions, was also observed in the LDs, the unsaturated/saturated FA ratio indicated a greater content of unsaturated FAs. Some polyunsaturated FAs observed in the LD analysis have distinct and contrasting effects in cancer: arachidonic acid mostly exhibits pro-tumourigenic effects [82]; eicosapentaenoic (EPA; 20:5, ω-3) and docosahexaenoic acids (DHA; 22:6, ω-3) possess anti-tumourigenic, anti-inflammatory, and pro-apoptotic effects in cancer cells [83].

The mitochondrial dysfunction observed in our studies does not support an active metabolic role for LDs and further suggests that other compensatory metabolic adaptations to support cell survival under conditions of iron starvation must be considered.

## Conclusions

Iron depletion in breast cancer cells induced mitochondrial dysfunction and inhibited the activities of several essential enzymes requiring iron as a cofactor. The morphological, proteomic, and metabolic aspects of our study support the view that cells develop a short-term stress tolerance under iron depletion, activating a hypoxia-like program with the uptake of nutrients from the extracellular fluid-phase by macropinocytosis. However, the blockage of autophagy and the dysfunctional mitochondrial respiration do not permit a sufficient level of catabolism to sustain long-term cell survival. Drastic cell structure changes guided by ER expansion create two main storage compartments: extracellular fluid-filled vacuoles and LDs that accumulate lipids because of the loss of FA oxidation. LDs released after cell death have potential benefits for study as metabolic entities to understand the mechanistic and clinical aspects of tumour biology in future studies.

## Additional files

Additional file 1: Table S1. List of primary antibodies used for western blot analysis. (DOCX 11 kb)

Additional file 2: FACS analysis of cell cycle. (PPTX 200 kb)

Additional files 3: Reports of statistical results, specifically up- and down- regulated proteins in DFO- or Dp44mT-treated cells with respect to control. Down-regulated in DFO- treated cells versus untreated. (XLS 618 kb)

Additional file 4: Up-regulated in DFO-treated cells versus untreated. (XLS 375 kb)

Additional file 5: Down-regulated in Dp44mT-treated cells versus untreated. (XLS 721 kb)

Additional file 6: Up-regulated in Dp44mT-treated cells versus untreated. Differential expression was considered as significant if (1) a protein was present only in untreated or treated cells or (2) its normalised (according to the LFQ algorithm) intensity resulted in a statistical difference, as calculated by the welch’s t-test (t-test cut-off at *p* value = 0.0167). These data have been deposited to the ProteomeXchange Consortium (http://proteomecentral.proteomexchange.org/cgi/GetDataset) via the PRIDE (Vizcaíno et al., 2016 PubMed ID: 26527722) partner repository with the dataset identifier PXD007595. (XLS 537 kb)

Additional file 7: Ingenuity Pathway analysis revealing the pathways significantly changed after the DFO/Dp44mT treatments. (PPTX 397 kb)

## Abbreviations

(PLIN) 2: Perilipin
ACACA: lipogenic acetyl-CoA carboxylase
ANOVA: Analysis of variance
ARA: Arachidonic acid
ATCC: American Type Culture Collection
BCA: Bicinchoninic acid
BCL-2: B-cell lymphoma 2
BHT: Butylated hydroxy toluene
CARS: Coherent anti-Stokes Raman scattering
DAPI: 4′,6-diamidino-2-phenylindole
DAPI: 4′,6-diamidino-2-phenylindole; differential interference contrast
DEGS: D4-dihydroceramide (DhCer) desaturase
DFO: Deferoxamine
DHA: Docosahexaenoic acids
DMEM: Dulbecco’s modified Eagle’s medium
DMSO: Dimethyl sulfoxide
Dp44mT: Di-2-pyridylketone 4,4-dimethyl-3-thiosemicarbazone
DpT: Di-2-pyridylketone thiosemicarbazone
EMT: Epithelial–mesenchymal transition
EPA: Eicosapentaenoic
ER: Endoplasmic reticulum
FA: Fatty acid
FACS: Fluorescence-activated cell sorting
FADS: FA desaturase
FADS2: delta-6 desaturase
FASN: FA synthase
FBS: Foetal bovine serum
FDR: False discovery rate
Fru-6P: Fructose-6-phosphate
GC-MS: Gas chromatography–mass spectrometry
GFPT1: Gln–fructose-6-phosphate transaminase 1
GO: Gene ontology
GOT: Glutamic-oxaloacetic transaminase
HBP: Hexosamine biosynthetic pathway
HIF: Hypoxia-inducible factor
HMGB1: High mobility group box 1
IF: Immunofluorescence
KEGG: Kyoto Encyclopedia of Genes and Genomes
LAMP 1: Lysosomal-associated membrane protein
LC3: microtubule-associated protein light chain 3 (LC3)
LD: Lipid droplets
LQF: Label-free quantification
LY: Lucifer yellow
LysoTracker: Lysosomotropic metachromatic fluorochrome
MCR-ALS: Multivariate Curve Resolution Alternating Least Squares
MTT: 3-[4,5-dimethylthiazol-2-yl]-2,5-diphenyltetrazolium bromide; thiazolyl blue
NDRG1: N-myc downstream-regulated gene 1
PARP: Poly (ADP-ribose) polymerase-1
ROS: Reactive oxygen species
RTN4: Reticulon 4
SCD: Stearoyl-CoA desaturase
SEM: Standard error of the mean
SLC1A5: Sodium-dependent neutral amino acid transporter type 2
SLIRP: SRA stem-loop interacting RNA-binding protein
STRING: Search Tool for the Retrieval of INteracting Genes/proteins
TCA: Tricarboxylic acid
TfR: Transferrin receptor
