# Supplementary material for: Lipid accumulation in human breast cancer cells injured by iron depletors
Source: J Exp Clin Cancer Res. 2018 Apr 3;37:75. doi: 10.1186/s13046-018-0737-z (PMC5883539; doi:10.1186/s13046-018-0737-z)
Supplement: Supplementary file 1 — Table S1. List of primary antibodies used for western blot analysis. (DOCX 11 kb) [file 13046_2018_737_MOESM1_ESM.docx]

**Additional file 1**

**Antibodies for Western blot**

Phospho-JNK (Thr183/Y182) purchased from Cell Signaling Technology, Inc.

AKT purchased from Cell Signaling Technology, Inc.

Phospho-AKT (Ser 473) purchased from Cell Signaling Technology, Inc.

ERK ½ purchased from Sigma-Aldrich.

Phospho-ERK ½ (Thr183/Tyr185) purchased from Sigma-Aldrich.

AMPKα purchased from Cell Signaling Technology, Inc.

Phospho-AMPKα (Thr172) purchased from Cell Signaling Technology, Inc.

mTOR purchased from Cell Signaling Technology, Inc.

Phospho-mTOR (Ser2448) purchased from Cell Signaling Technology, Inc.

HGMB1 purchased from Sigma-Aldrich.

PARP cleaved purchased from Cell Signaling Technology, Inc.

LC3B purchased from Cell Signaling Technology, Inc.

Bcl-2 purchased from Cell Signaling Technology, Inc.

β−Actin purchased from Sigma-Aldrich.

β−Tubulin purchased from Sigma-Aldrich.

NDRG1 purchased from Sigma-Aldrich.

RTN4 purchased from Sigma-Aldrich.

Phospho-P38 purchased from New England Biolabs.
