# Supplementary material for: Lipid accumulation in human breast cancer cells injured by iron depletors
Source: J Exp Clin Cancer Res. 2018 Apr 3;37:75. doi: 10.1186/s13046-018-0737-z (PMC5883539; doi:10.1186/s13046-018-0737-z)
Supplement: Supplementary file 2 — FACS analysis of cell cycle. (PPTX 200 kb) [file 13046_2018_737_MOESM2_ESM.pptx]

## Slide 1
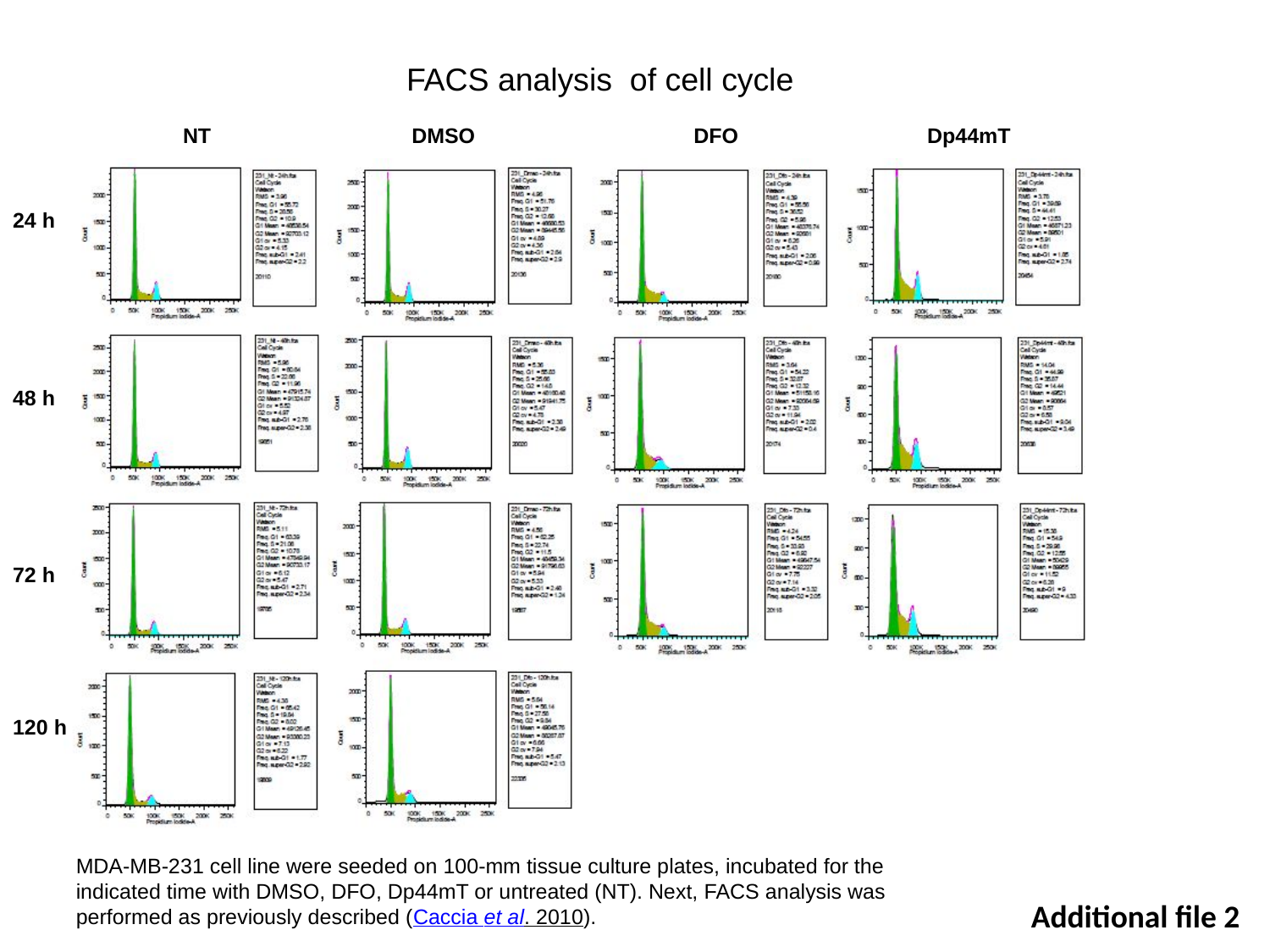

FACS analysis of cell cycle
NT DMSO DFO Dp44mT
24 h
48 h
72 h
120 h
MDA-MB-231 cell line were seeded on 100-mm tissue culture plates, incubated for the indicated time with DMSO, DFO, Dp44mT or untreated (NT). Next, FACS analysis was performed as previously described (Caccia et al. 2010).
Additional file 2
