# Supplementary material for: Lipid accumulation in human breast cancer cells injured by iron depletors
Source: J Exp Clin Cancer Res. 2018 Apr 3;37:75. doi: 10.1186/s13046-018-0737-z (PMC5883539; doi:10.1186/s13046-018-0737-z)
Supplement: Supplementary file 7 — Ingenuity Pathway analysis revealing the pathways significantly changed after the DFO/Dp44mT treatments. (PPTX 397 kb) [file 13046_2018_737_MOESM7_ESM.pptx]

## Slide 1
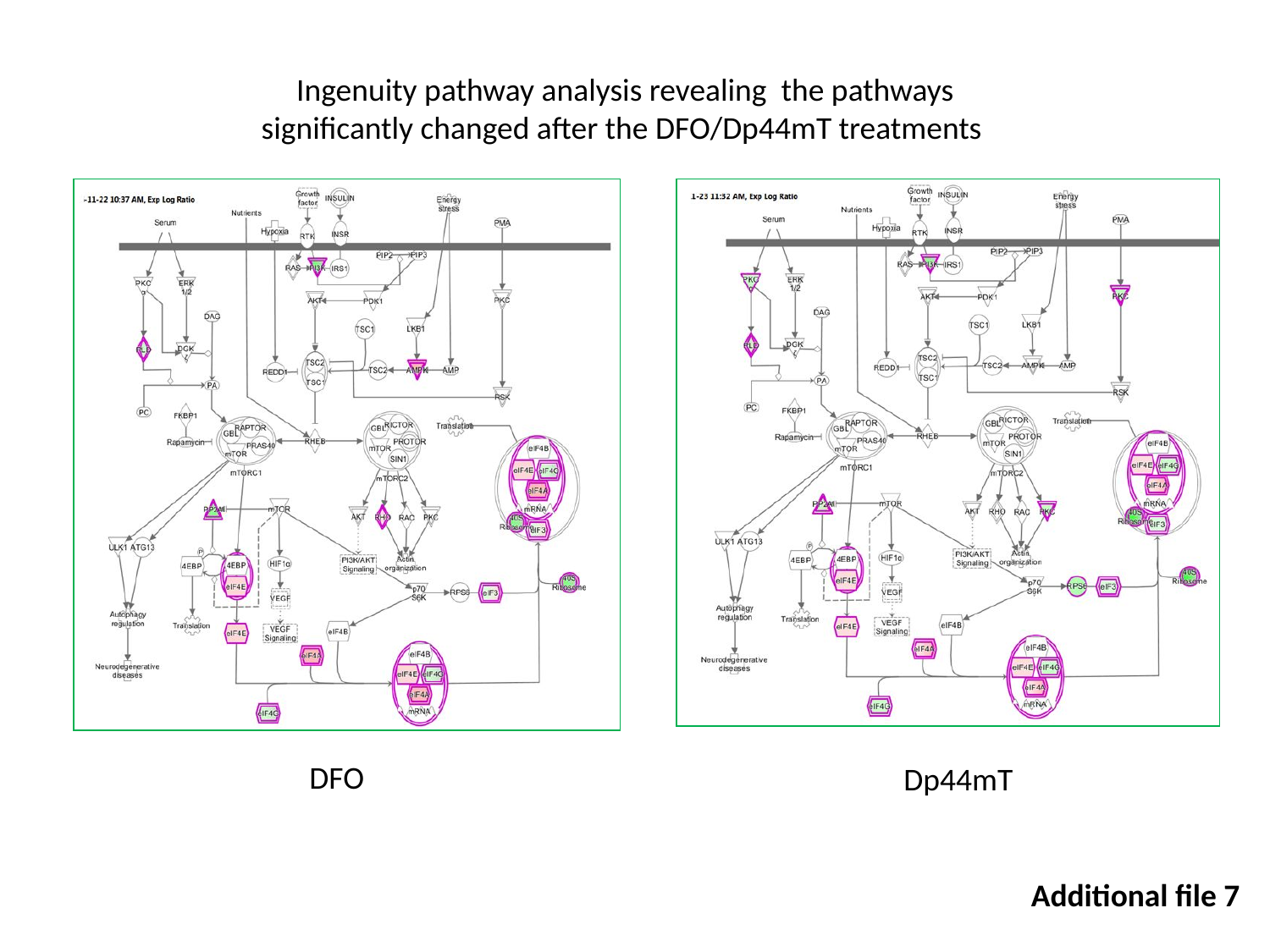

Ingenuity pathway analysis revealing the pathways
significantly changed after the DFO/Dp44mT treatments
DFO
Dp44mT
Additional file 7
